# Phospholipase PLA2G5-triggered hemolysis emerges as a contributor to sepsis lethality

**DOI:** 10.1172/JCI205822

**Published:** 2026-05-01

**Authors:** Jean-Marc Cavaillon

**Affiliations:** Institut Pasteur, Paris, France.

## Abstract

Despite extensive advances in understanding sepsis pathophysiology, treatment outcomes have not substantially improved. In this issue, Takahama and colleagues identified phospholipase A2 Group V (PLA2G5) as a contributor to sepsis lethality in mouse models of endotoxemia and sepsis. Whole-mouse spatial profiling generated bodywide maps of systemic inflammation and uncovered intestinal goblet cells as a source of pathogenic PLA2G5. Pairs of inflammatory cytokines (TNF and IFN-γ, or TNF and IL-18) induced PLA2G5 expression in goblet cells. Mechanistically, circulating PLA2G5 triggered intravascular hemolysis through its lipolytic activity on erythrocyte membranes and contributed to organ failure and death. PLA2G5’s deleterious effects were blocked by specific antibodies and were absent in *Pla2g5-*deficient mice. In humans with bacterial or fungal sepsis or severe COVID-19, plasma PLA2G5 levels were elevated and predicted disease severity. This discovery highlights the contribution of hemolysis to sepsis, suggesting that PLA2G5 inhibitors, hemoglobin, or heme antagonists could represent valuable therapeutic tools.

## Sepsis remains to be fully understood

The global incidence of sepsis remains unacceptably high. The most recent estimates indicate an annual worldwide burden of 166 million cases and 21.4 million sepsis-related deaths in 2021 (1). In response to this major health challenge, the World Health Organization (WHO) adopted a resolution aimed at improving the prevention, diagnosis, and management of sepsis (2). Despite tremendous efforts to decipher the molecular and cellular mechanisms underlying sepsis, accumulated knowledge has not yet led to innovative or effective new therapies (3). One major advance in recent decades is the recognition that the population of individuals affected by sepsis is highly heterogeneous — not only in terms of gender, age, infectious agents, and infection sites, but also in terms of each individual’s intrinsic responsiveness to interventions. Accordingly, identifying patient subpopulations based on endotypes may help improve treatment responses, as recently illustrated by a personalized approach (4).

## Sepsis: an adapted overwhelming inflammation

Sepsis is associated with multiple organ failure (MOF) resulting from excessive systemic inflammation (5). Although the current official definition describes sepsis as a dysregulated host response to infection, this concept may be flawed, as evolution has shaped the immune system to respond optimally to infection rather than in a dysregulated manner. “Overwhelming inflammation” may be a more precise characterization of sepsis. It implies the simultaneous release of both pro- and antiinflammatory mediators (6), and implies that all tissues mount their own specific responses in a compartmentalized manner initially aimed to fight the infectious agents and then to restore homeostasis. In prior work that measured dynamic gene-expression changes across organs in a murine sepsis model or in mice undergoing systemic inflammation after lipopolysaccharide (LPS) injection, Takahama et al. (7) showed that pairwise combinations of TNF with IL-18, IFN-γ, or IL-1β are sufficient to recapitulate the transcriptomic impact of endotoxemia and sepsis across tissues. These findings highlighted the central role of inflammatory cytokines and their synergistic effects during sepsis.

## Sepsis and hemolysis

Hemolysis is frequently observed in sepsis patients. It may result from bacterial hemolysins or host-mediated erythrocyte destruction. Measurements of anemia, hemolysis, cell-free hemoglobin, and heme have consistently been associated with sepsis severity and outcomes (8, 9). Hemolysis further amplifies inflammation. For example, hemoglobin infusion in mice enhances TNF release in response to LPS, and Kupffer cells from these mice produce more TNF in vitro than those from control mice (10). Hemoglobin administered intravenously before, during, or after LPS injection markedly increases lethality in a dose-dependent manner (11). Heme is also pathogenic, as it catalyzes the formation of reactive oxygen species, oxidizing lipids and proteins, damaging DNA, and causing cell and tissue injury (12). Moreover, heme plays a central role in severe sepsis by inducing coagulation in a tissue factor–dependent manner (13), activating the NLRP3 inflammasome and promoting IL-1β release (14), and driving sepsis-associated cardiac endothelial senescence (15).

## PLA2G5, hemolysis, and the gut

**Figure 1:**
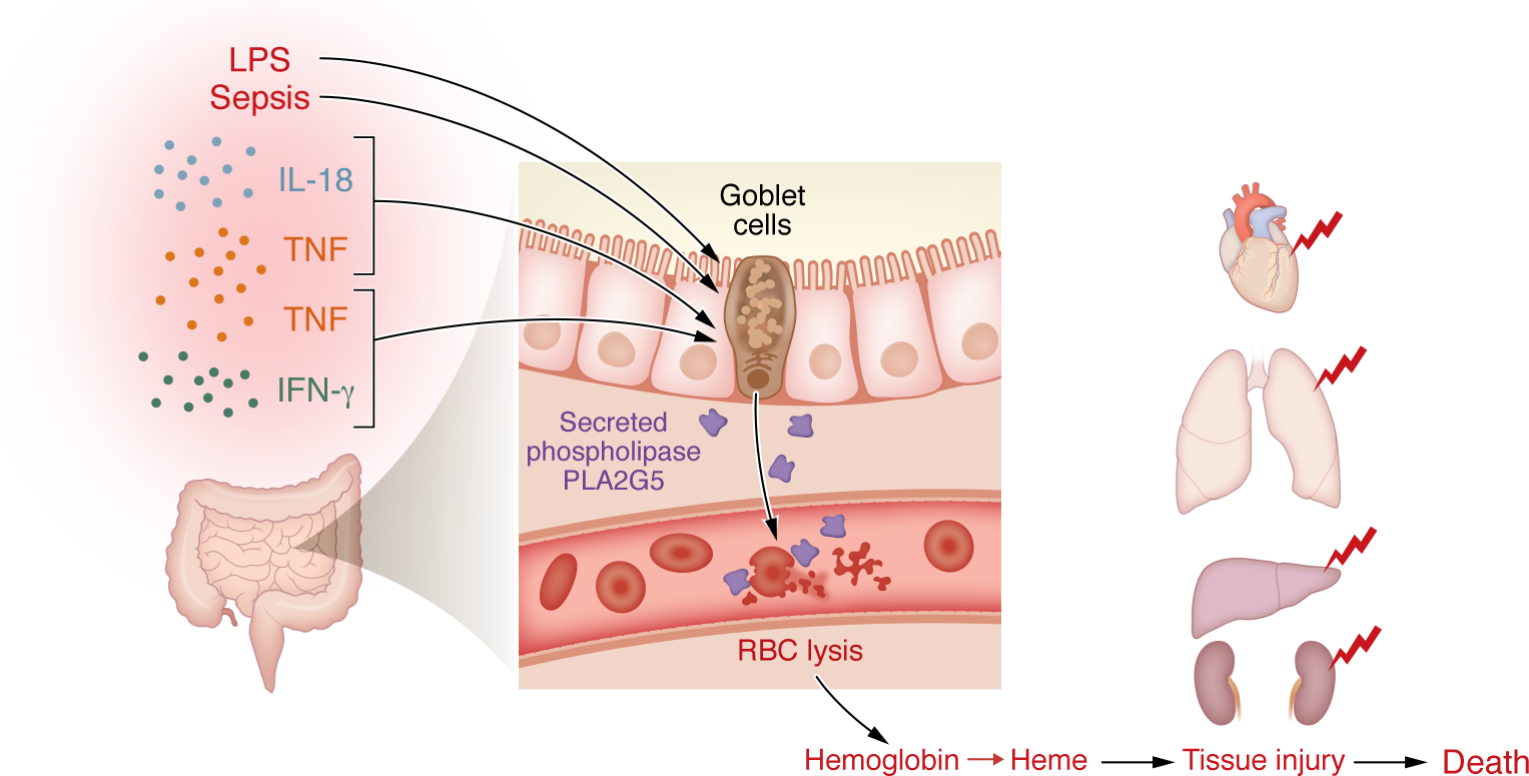
Contribution of secreted phospholipase A2 Group V (PLA2G5) to red blood cell lysis during sepsis. In mouse models, Takahama et al. (7) showed that sepsis or endotoxemia — mimicked by specific pairs of inflammatory cytokines — induced the production of PLA2G5 by goblet cells within the gut. Once released into the bloodstream, PLA2G5 triggered erythrocyte lysis, leading to the release of hemoglobin and heme, which contributed to organ failure and eventually death. Elevated PLA2G5 levels in humans with bacterial or fungal sepsis or severe COVID-19 predicted disease severity, supporting translation of these findings to clinical understanding of sepsis.

Many mechanisms have been proposed to explain hemolysis during sepsis (16), but no direct mediator has ever been identified. Takahama and colleagues (17) now report the discovery of phospholipase A2 Group V (PLA2G5) as a key driver of hemolysis associated with sepsis and endotoxemia. Using whole-mouse spatial transcriptomics, they found that *Pla2g5* gene expression was upregulated in the colon and small intestine after LPS injection and in two murine sepsis models: cecal ligation and puncture (CLP) and cecal slurry injection. The consistent identification of PLA2G5 in two distinct sepsis models that are known to produce markedly different transcriptomic profiles (18) strengthens the robustness of their findings. PLA2G5 exerted its harmful effects by inducing erythrocyte lysis through hydrolysis of membrane phospholipids, as demonstrated using recombinant PLA2G5. Notably, phospholipases from bacteria (19) and snake venoms (20) are also known to induce hemolysis. Using anti-PLA2G5 antibodies and PLA2G5-deficient mice, Takahama et al. showed that PLA2G5 contributed to hypothermia, MOF (as assessed by renal [blood urea nitrogen], hepatic [alanine aminotransferase] and cardiac [troponin I] biomarkers), and lethality. PLA2G5 was present at baseline in several tissues, particularly the heart, spleen, and kidney, and its expression increased in gut goblet cells and colonic secretory cells after LPS injection and in sepsis models (Figure 1). These findings echo the long-standing concept of the “gut as the motor of MOF,” proposed by Charles James Carrico (1935–2002), who suggested that impaired gastrointestinal epithelial barrier function and bacterial translocation fuel systemic inflammation (21). The discovery by Takahama et al. offers a fresh perspective on the gut’s contribution to sepsis and MOF.

Takahama and colleagues next analyzed samples from patients hospitalized with bacterial and fungal sepsis, as well as severe COVID-19, and determined that serum PLA2G5 levels were higher in these patients than in samples from hospitalized patients without sepsis or patients discharged from emergency departments (17). These data suggest that gut-derived PLA2G5 expression would be restricted to severe infectious conditions. Higher PLA2G5 levels correlated with elevated Sequential Organ Failure Assessment (SOFA) scores and predicted patient outcomes. The authors also showed that PLA2G5 does not influence pro- or anti-inflammatory cytokine levels but modulates splenic red pulp macrophages and iron homeostasis.

Why would evolution preserve an enzyme with harmful effects? Like many inflammatory mediators, PLA2G5 exhibits a yin-yang duality. Identified in 1994, this phospholipase hydrolyzes membrane phospholipids to generate lysophospholipids and free fatty acids and can exert both protective and detrimental functions (22). Produced by many other cell types, including macrophages, epithelial cells, dendritic cells, cardiomyocytes, neutrophils, adipocytes, and pancreatic β-cells, PLA2G5 is implicated in allergic asthma, arthritis, and cardiovascular diseases. Conversely, it contributes to host defense against fungal and bacterial pathogens by promoting phagocytosis, leukocyte recruitment, and pathogen clearance (22). It also induces the release of angiogenic factors (23), and antiviral activities have been reported for phospholipases (24).

Several questions arise from these findings. What is the significance of the high baseline expression of *Pla2g5* gene in the spleen, kidney, and heart of control mice, and why does its high expression in these tissues not cause the same deleterious effects observed when the gene transcription is induced in the gut after LPS or CLP? One hypothesis is that compartmentalization governs distinct local versus systemic consequences. Remarkably, deletion of *Pla2g5* or treatment with antiPLA2G5 antibodies did not affect cytokine expression or lipid metabolites during systemic inflammation. Given the proinflammatory properties of hemoglobin and heme released following PLA2G5-mediated hemolysis, these surprising results may reflect the timing of measurements. However, in multitissue gene-expression analyses of mice injected with LPS and anti-PLA2G5, the authors showed that blocking this phospholipase reduced the expression of several regulatory molecules across tissues, including a metalloproteinase inhibitor, plasminogen activator inhibitor-1, and immune checkpoint inhibitors (CTLA-4, PD-1, and PDL-1). These latter molecules are known contributors to the immune dysregulation frequently observed in patients with sepsis. Accordingly, these observations suggest that PLA2G5 may influence not only inflammation but also its counterregulatory pathways.

## Conclusions

Distinct inflammatory pathways are activated during sepsis, affecting tissues differently. Within the bloodstream, localized phenomena such as coagulation and hemolysis occur. The difficulty in developing new therapies that improve sepsis outcomes likely reflects the extensive synergy among inflammatory mediators, cascades of signaling, and mechanisms activated during sepsis. The discovery of gut-derived PLA2G5 as a driver of sepsis-associated erythrocyte lysis, tissue injury, poor outcomes in experimental endotoxemia and sepsis, and elevated serum PLA2G5 levels in patients with severe sepsis brings renewed attention to hemolysis in sepsis. These findings should encourage new clinical trials aimed at antagonizing PLA2G5, neutralizing free hemoglobin (e.g., with haptoglobin), or neutralizing heme (e.g., with hemopexin) (25, 26). Given that patients with septic shock exhibit significantly reduced levels of haptoglobin and hemopexins compared with patients with sepsis (27), measuring hemolysis and its accompanying biomarkers may help refine precision medicine approaches.

## Conflict of interest

The author has declared that no conflict of interest exists.

## Version 1 05/01/2026

Electronic publication
